# Influenza vaccination for heart failure patients: a cost-effectiveness analysis from the perspective of Chinese healthcare system

**DOI:** 10.3389/fpubh.2024.1348207

**Published:** 2024-08-09

**Authors:** Minting Zhao, Fuqiang Liu, Lan Wang, Dan Chen

**Affiliations:** ^1^School of Art & Design, Shaanxi University of Science and Technology, Xi’an, China; ^2^School of Biological and Pharmaceutical Science, Shaanxi University of Science and Technology, Xi’an, China; ^3^Cardiovascular Department, Shaanxi Provincial People's Hospital, Xi’an, China

**Keywords:** influenza vaccine, cost-effectiveness analysis, heart failure, cost-utility analysis, influenza infection

## Abstract

**Purpose:**

Influenza infection induces cardiovascular events in heart failure (HF) patients, with potential risk reduction through vaccination. This study aims to evaluate the cost-effectiveness of influenza vaccination for HF patients in China.

**Methods:**

We developed a Markov model with a 3-month cycle to simulate the cost-effectiveness of administering the influenza vaccine to patients with HF over a 3-year period. Patients in the model received either the influenza vaccine or a placebo, in addition to standard HF treatment. Cost data, sourced from the China Healthcare Statistic Yearbook and other public records, and effectiveness data from the IVVE (Influenza Vaccine to Prevent Adverse Vascular Events in HF) trial, were incorporated. Specifically, the cost of the influenza vaccine was 75 Chinese Yuan (CNY) (11 USD), the cost of hospitalization for heart failure (HHF) was 9,326 CNY (1,386 USD), and the cost of treatment for pneumonia was 5,984 CNY (889 USD). The study’s primary outcome, the incremental cost-effectiveness ratio (ICER), quantifies the incremental cost (CNY and USD) per incremental quality-adjusted life year (QALY). Additional outcomes included total cost, total effectiveness, incremental cost, and incremental effectiveness. We conducted one-way and probabilistic sensitivity analyses (PSA) to assess certainty and uncertainty, respectively. Scenario analysis, considering various situations, was performed to evaluate the robustness of the results.

**Results:**

In the base case analysis, influenza vaccine, compared to placebo, among Chinese HF patients, resulted in a cost increase from 21,004 CNY (3,121 USD) to 21,062 CNY (3,130 USD) and in QALYs from 1.89 to 1.92 (2.55 life years vs. 2.57 life years) per patient. The resulting ICER was 2,331 CNY (346 USD) per QALY [2,080 CNY (309 USD) per life year], falling below the willingness-to-pay threshold based on per capita GDP. One-way sensitivity analysis revealed that disparities in HHF and cardiovascular death rates between groups had the most significant impact on the ICER, while the cost of vaccines had a marginal impact. PSA and scenario analysis collectively affirmed the robustness of our findings.

**Conclusion:**

This study suggests that adding the influenza vaccine to standard treatment regimens for Chinese patients with HF may represent a highly cost-effective option. Further real-world data studies are essential to validate these findings.

## Introduction

Heart failure (HF) is a clinical syndrome characterized by structural and/or functional abnormalities of the heart (1), and HF patients exhibit an elevated propensity for vascular adverse events and other critical comorbidities (2). Although mortality rates have declined in recent years, the incidence of HF is increasing globally, placing a heavy burden on healthcare systems (3), especially in developing country. For instance, the age-standardized prevalence of patients with HF aged 25 years and above in China was recorded as 1.10% in 2017, accounting for a total of 12.1 million patients, and the annual cost per-capita for inpatient and outpatient amounts to $4,406.8 and $892.3, respectively (4).

Influenza infection is considered one of the inducing factors for cardiovascular (CV) events in HF patients, augmenting the risk of CV-related mortality, all-cause death, and hospitalization (5, 6). Hence, influenza vaccination may mitigate the risk theoretically, which was also recommended by guideline for decades (7). However, the current clinical evidence remains insufficient. Two meta-analyses suggest that influenza vaccination may reduce the overall mortality risk in HF patients, and one of them found it can also lower CV-related mortality, but both of the included studies were in low certainty of evidence (8, 9).

Furthermore, a recent multicenter, randomized, double-blinded, placebo-controlled trial investigated the efficacy of the influenza vaccine in HF patients across 30 international centers, including six in China, which contributed 13.5% of the total study population, making it the third largest participating country (10). The trial demonstrated that vaccination reduced all-cause hospitalizations (hazard ratio [HR] 0.84, 95% confidence interval [CI] 0.74–0.97, *p* = 0.013) and pneumonia incidence (HR 0.58, 95% CI 0.42–0.80, *p* = 0.0006). Although no significant difference was observed in terms of cardiovascular (CV) events, almost all outcomes in the influenza vaccine group were lower than those in the placebo group. Additionally, studies found that for the older adult, influenza vaccination is associated with direct cost savings and reduced hospitalization rates (11, 12). Therefore, administering the influenza vaccine to HF patients could potentially offer economic advantages as an affordable and straightforward intervention by reducing expenses related to all-cause hospitalizations, incidence of pneumonia, and CV events.

Particularly in China, despite recommendations from the local guidelines, the influenza vaccination rates remain significantly low in most cities (13). Therefore, a cost-effective analysis on the expense in influenza vaccination is needed. Based on the trial, this study aims to develop a mathematical model and assess the cost and effectiveness of influenza vaccination for HF patients in China.

## Methods

### Model overview

We developed a Markov model to assess the cost-effectiveness for Chinese patients with HF. This Markov model has found widespread application in the pharmacoeconomic evaluation of HF patients (14–16). In simple terms, the model comprised five health states: “New York Heart Association (NYHA) I,” “NYHA II,” “NYHA III,” “NYHA IV” and “Dead.” In our study, patients entered the Markov model starting from the same initial health state as in the IVVE (Influenza Vaccine to Prevent Adverse Vascular Events) trial (10), a randomized controlled trial (RCT) investigating the efficacy of influenza vaccine in HF patients. Patients in the Markov model could experience one of five events in each Markov cycle: “Hospitalization for HF (HHF),” “Pneumonia,” “CV death,” “non-CV death” or “No event.” It was assumed that patients who experienced ‘No event’ had the possibility to transition to a better, worse, or remain in the same NYHA classification during the subsequent Markov cycle. However, those who experienced ‘HHF’ could not transition to a better NYHA classification in the subsequent cycle. For example, if a patient was at a NYHA II state and experienced an HHF in the current Markov cycle, they could transition to NYHA II or NYHA III but not to NYHA I in the subsequent 3 months. However, this does not mean they could never transition to NYHA I; they could do so if their HF condition remained stable for 3 months (a Markov cycle). This assumption was in line with common clinical practice and was also used in other health technology assessments for HF treatment (16, 17). Furthermore, patients who experienced CV or non-CV death were directly transitioned to the “Dead” state, effectively exiting the Markov model.

**Figure 1:**
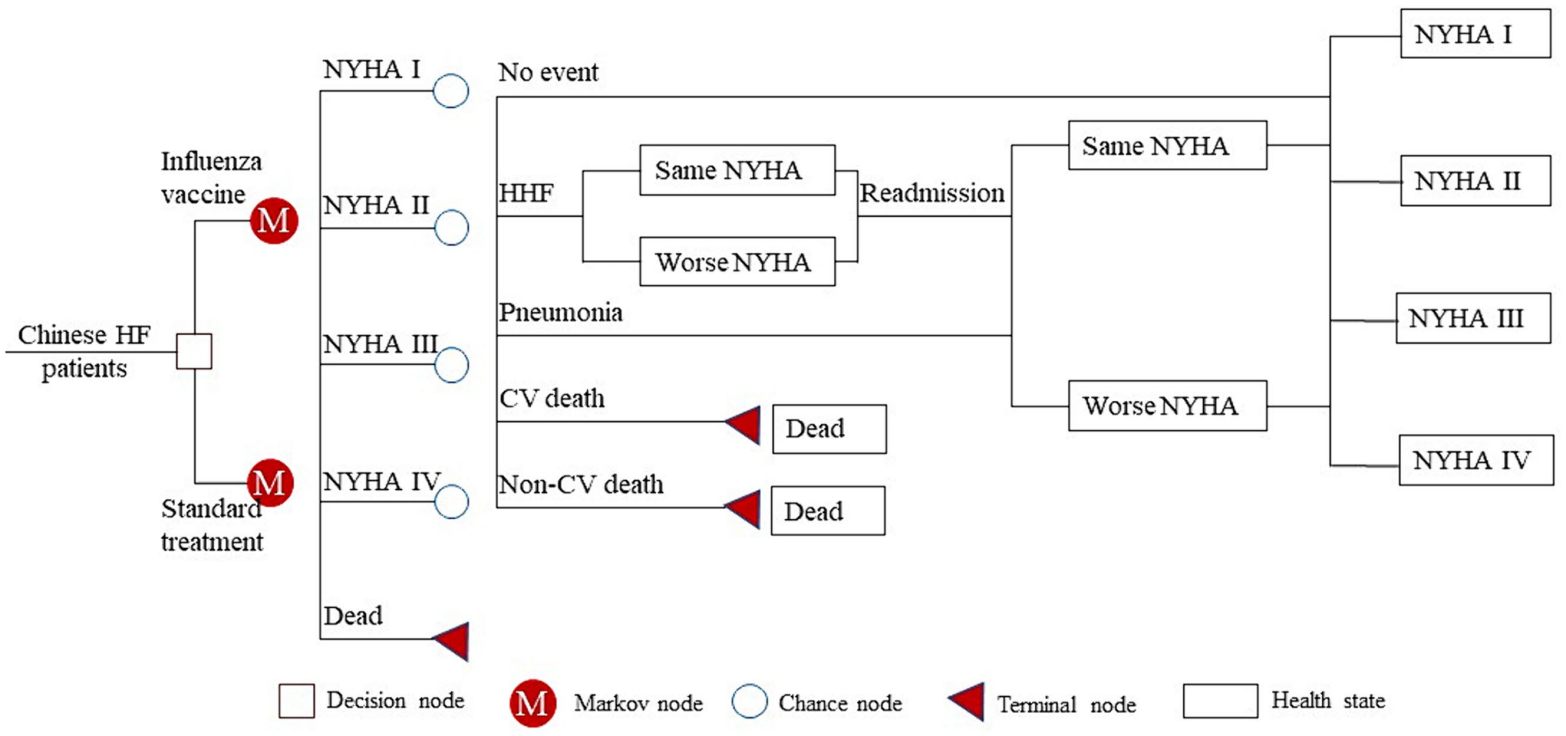
Schematic diagram of the Markov model. HF, heart failure; NYHA, New York Heart Association; HHF, hospitalization for heart failure; CV, cardiovascular.

Our Markov model simulated the 3-year cost and effectiveness for Chinese HF patients, with a cycle length of 3 months. The schematic diagram of the Markov model is shown in Figure 1. Model building and analyses were performed with TreeAge Pro 2022 (Williamstown, MA, USA).

### Intervention and control

Over three consecutive influenza seasons, participants were administered either a 0.5 mL dose of inactivated influenza vaccine, or a placebo (saline), intramuscularly once a year. The trivalent vaccine (TIV) consisted of 15 μg of haemagglutinin per 0.5 mL dose for each of the two influenza type A subtypes (H1N1 and H3N2), as well as for an influenza type B/Brisbane/60/2008-like virus. Notably, in China, the influenza season typically spans from November to March. Instead of administering the influenza vaccine within the same month each year, participants were given the option to receive it on the appropriate date aligned with the local influenza season.

Participants in both groups received standard treatment for HF, in addition to either the influenza vaccine or a placebo. The standard treatment regimen for HF outlined in the China National Heart Failure Guideline 2023 encompasses a multifaceted approach, primarily revolving around several key medications. These include angiotensin converting enzyme inhibitors, angiotensin receptor blockers, angiotensin receptor neprilysin inhibitors, β blockers, diuretics, mineralocorticoid receptor antagonists, and sodium and glucose cotransporter-2 inhibitors. Additionally, for specific HF subtypes, adjunctive therapies such as digoxin and soluble guanylate cyclase stimulators may also be prescribed.

### Population

The study population comprised Chinese HF patients with baseline characteristics akin to those in the IVVE trial (10). In the IVVE trial, patients had a mean age of 57.2 years, with 51.4% being female, and were predominantly classified as NYHA II (69.5%), with lesser proportions in NYHA III (26.1%) and IV (4.4%). HF with preserved ejection fraction (EF, HFpEF) was observed in 22.6% of patients, while the remaining 77.4% had HF with mildly reduced EF (HFmrEF) or HF with reduced EF (HFrEF). Comparatively, in a study exploring HF prevalence in China, Chinese patients exhibited moderate differences. They were older (63.9 ± 13.2 vs. 57.2 ± 15.3 years) and had higher systolic blood pressure (137.2 ± 22.3 vs. 125.7 ± 23.7 mm Hg) but demonstrated a lower prevalence of hypertension (55.3% vs. 64.9%). However, heart rate, gender distribution, left ventricular function, diabetes, and atrial fibrillation were similar between the cohorts (18).

The patients were randomized to allocate to either the influenza vaccine group or the placebo group in a 1:1 ratio. Regardless of their allocation, all patients received standard treatment. In the influenza vaccine group, patients received an annual intramuscular dose of 0.5 mL of inactivated influenza vaccine, which was recommended for the specific influenza season. Alternatively, patients in the placebo group received a saline injection. It’s important to note that the vaccine used during the trial was trivalent, but it is replaced by a quadrivalent vaccine (QIV) currently (10, 19).

### Input parameters

#### Transition probability

The transition probabilities for HHF and CV death were directly obtained from the IVVE trial (10). These probabilities were converted from incidence rates to 3-month transition probabilities using the formula: ‘3-month transition probability = 1 − exp (−3-month rate).’ The 3-month rate was calculated using the formula: ‘3-month rate = −ln (1 − incidence rate)/Period number (Table 1). Employing the aforementioned formulas, we calculated that the 3-month rate for HHF in the vaccine group was 0.015195761, determined as −ln (1 − 334/2,560)/27.6*3, where 334 represented the HHF events in the vaccine group, 2,560 represented the total patients in the vaccine group, 27.6 represented the follow-up period, and 3 represented the cycle length (10). Subsequently, we obtained the 3-month transition probability for HHF, which was 0.015080888, calculated as 1 − exp (−0.015195761), where 0.015195761 represented the 3-month rate for HHF. Similarly, we derived the remaining transition probabilities for HHF and CV death (Supplementary material).

**Table 1:** Input parameters in the Markov model.

Parameters	Base case	Range	Source
Transition probabilities (per Markov cycle)			
HHF in vaccine^a^	0.0109	0.0095–0.0122	Ref. (10)
HHF in ST^a^	0.0123	0.0109–0.0138	Ref. (10)
CV death in vaccine^a^	0.0151	0.0135–0.0167	Ref. (10)
CV death in ST^a^	0.0170	0.0153–0.0187	Ref. (10)
Pneumonia in vaccine^a^	0.0026	0.0020–0.0033	Ref. (10)
Pneumonia in ST^a^	0.0045	0.0036–0.0053	Ref. (10)
Non-CV mortality of general population			
55–59 years old	0.0032	/	Ref. (20)
60–64 years old	0.0046	/	Ref. (20)
65–69 years old	0.0074	/	Ref. (20)
70–74 years old	0.0115	/	Ref. (20)
75–79 years old	0.0180	/	Ref. (20)
RR of non-CV death in HF patients	2.50	1.61–4.00	Ref. (21)
Costs, CNY (USD)			
HHF (per time)	9,326 (1,386)	4,663–13,989	Ref. (20)
ST (per year)	7,011 (1,042)	3,506–10,517	Ref. (4)
Pneumonia	5,984 (889)	2,842–13,054	Ref. (22)
Vaccine (per dose)	75 (11)	46–138	Ref. (23)
Administration (per time)	20 (3)	5–40	Local data
Utilities			
NYHA I	0.732	0.695–0.769	Ref. (24)
NYHA II	0.78	0.741–0.819	Ref. (24)
NYHA III	0.715	0.679–0.751	Ref. (24)
NYHA IV	0.66	0.627–0.693	Ref. (24)
HHF or readmission	−0.1	0.08–0.13	Refs. (14–17)
Discount rate in China	0.05	0–0.08	Ref. (25)

HHF, hospitalization for heart failure; ST, standard treatment; CV, cardiovascular; RR, risk ratio; HF, heart failure; CNY, Chinese Yuan; NYHA, New York Heart Association.

^a^The calculation method is detailed in the Supplementary materials.

For the transition probability of non-CV death, it was calculated by multiplying the risk ratio (21), which represented the increased risk of non-CV death in HF patients compared to the general population of the same age, with the background mortality of the general population at the same age (20) (Table 1). The background mortality data for the general population was sourced from the China Health Statistical Yearbook 2022, publicly available (20).

The transition probabilities between NYHA classifications were accessed from a published study (16) (Table 2). Although there are no patients in the NYHA I state at the initial health state in the Markov model, patients in the NYHA II state have a chance to transition to the NYHA I state in subsequent cycles. Therefore, the health state of NYHA I was included in the Markov model, and its utility was also incorporated into the analysis.

#### Cost

The TIV was priced at 75 Chinese Yuan (CNY) (11 USD), while the QIV costed 138 CNY (21 USD). These prices were determined by the rates negotiated with vaccine manufacturers during collective purchasing by the Chinese government, accurately reflecting the actual cost of influenza vaccines for most Chinese people (23). In our base case analysis, we used the cost of the TIV because the QIV was not available in China at the time of the IVVE trial. Additionally, there was a 20 CNY (3 USD) cost for vaccine administration (Table 1).

When considering HF-related costs, we included the costs associated with HHF and standard HF treatment. The cost of HHF in China, sourced from the China Healthcare Statistic Yearbook, was reported as 9,326 CNY (1,386 USD) per occurrence, representing the comprehensive costs within the country (20). The cost of standard HF treatment was derived from a national survey of over 50 million individuals, which aimed to investigate the prevalence and economic burden of HF in China. According to the survey, the annual cost of standard HF treatment was 892.3 USD in 2016, which, after adjusting for the exchange rate and healthcare consumer price index (CPI) in China, equated to 7,011 CNY (1,042 USD). The CPI values for the years 2015–2022 were: 1.027, 1.038, 1.06, 1.043, 1.024, 1.018, 1.004, and 1.006, respectively (20). For pneumonia treatment, the total cost for community-acquired pneumonia was reported as 5,683 CNY (844 USD), which inflated to 5,984 CNY (889 USD) in 2022 (22). Costs before 2022 were converted to 2022 values using the healthcare CPI, and future costs were discounted at a rate of 5% (range: 0–8%), according to the China Guidelines for Pharmacoeconomic Evaluations (25).

#### Utility

We obtained the health-related quality of life (HRQoL) values for HF patients from a Chinese domestic study (24). This study determined that the HRQoL utility scores for patients categorized by NYHA functional class were as follows: 0.732 for NYHA I, 0.78 for NYHA II, 0.715 for NYHA III, and 0.66 for NYHA IV. Additionally, the disutility associated with HHF or readmission was recorded as −0.1, a value commonly utilized in published research (15, 16) (Table 1).

#### Outcome

The primary outcome of the study was the incremental cost-effectiveness ratio (ICER), which represents the incremental cost per incremental effectiveness (measured in quality-adjusted life year, QALY). In the absence of a specific willingness-to-pay (WTP) threshold recommended by the Chinese government, we followed the guidance provided in the China Guidelines for Pharmacoeconomic Evaluations (25), which aligned with the recommendations of the World Health Organization (WHO). In this context, the influenza vaccine was deemed highly cost-effective when the ICER fell below the per capita gross domestic product (GDP), cost-effective if it was between one to three times the per capita GDP, and not cost-effective if it exceeded three times the per capita GDP (2022). The per capita GDP in China stood at 85,698 CNY (12,734 USD) in 2022. Secondary outcomes encompassed total cost, total effectiveness, incremental cost, and incremental effectiveness.

In our base case analysis, we set the starting age at 57 years old to align with the average age in the IVVE trial. Additionally, in the scenario analysis, we considered starting ages of 65, to align with the average age of HF patients in the China Hypertension Survey. Furthermore, we conducted additional scenario analyses to test the robustness of the results. These scenarios involved evaluating the cost-effectiveness of the vaccine in HF patients at the highest price of point and considering an alternative scenario where the HHF rate from the Urban Employee Basic Medical Insurance (UEBMI) scheme was used instead of the data from the IVVE trial (4).

### Sensitivity and scenario analysis

We conducted a one-way sensitivity analysis to evaluate how individual input parameters influenced the ICER. During this analysis, parameters were systematically varied within their 95% CI or predefined ranges. Specifically, for transition probabilities and utilities, we calculated and incorporated the 95% CI into the sensitivity analysis. Meanwhile, for the cost of the vaccine, we utilized the highest and lowest reported values as the range. Regarding the cost of HHF per time and annual standard treatment cost, due to the absence of reported CI or ranges, we employed 0.5 times and 1.5 times the reported costs as lower and higher bounds, respectively. These values were sourced from reputable references such as the China Healthcare Statistic Yearbook or national surveys, aiming to encompass the typical cost spectrum for Chinese HF patients. The results of the one-way sensitivity analysis were visually represented using a tornado diagram. For the probabilistic sensitivity analysis (PSA), we performed 10,000 Monte Carlo simulations to assess the robustness of our findings. In this analysis, all cost-related parameters were modeled with a gamma distribution, while transition probabilities and utilities were modeled with a beta distribution. Additionally, the relative risk (RR) of non-CV death in HF patients compared to the general population followed a log-normal distribution. The results of the PSA were presented through a scatter plot and a cost-effectiveness acceptability curve.

**Table 2:** Transition probabilities of NYHA classifications in the Markov model (every 3 months).

From to	NYHA I	NYHA II	NYHA III	NYHA IV
NYHA I	0.977	0.019	0.004	0
NYHA II	0.008	0.981	0.010	0.001
NYHA III	0	0.034	0.960	0.006
NYHA IV	0	0	0.055	0.945

## Results

### Base case analysis

In our base case analysis, after a 3-year simulation, each Chinese HF patient in the cohort would accumulate approximately 21,004 CNY (3,121 USD) in costs, resulting in an effectiveness of 1.89 QALYs (2.55 life years) with standard treatment. Alternatively, when administering the influenza vaccine alongside standard treatment, the cost would increase slightly to 21,062 CNY (3,130 USD), resulting in an effectiveness of 1.92 QALYs (2.57 life years) within the vaccine group. Comparing the inclusion of the influenza vaccine to standard treatment alone, the ICER amounted to 2,331 CNY (346 USD) per QALY [or 2,080 CNY (309 USD) per life year], which fell below the WTP threshold based on per capita GDP (Table 3).

**Table 3:** Results of base case and scenario analysis.

Strategy	Total cost, CNY (USD)	Incremental cost, CNY (USD)	Total effectiveness, QALY/LY	Incremental effectiveness, QALY/LY	ICER, CNY (USD) per QALY	ICER, CNY (USD) per LY
**Base case**						
Standard treatment	21,004 (3,121)		1.89/2.55			
Vaccine + standard treatment	21,062 (3,130)	58 (9)	1.92/2.57	0.02/0.03	2,331 (346)	2,080 (309)
**Scenario 1: Cost of influenza vaccine at the highest price**						
Standard treatment	21,004 (3,121)		1.89/2.55			
Vaccine + standard treatment	21,224 (3,130)	220 (33)	1.92/2.57	0.02/0.03	8,869 (1,318)	7,914 (1,176)
**Scenario 2: HHF rate from UEBMI**						
Standard treatment	34,668 (5,151)		1.75/2.55			
Vaccine + standard treatment	33,476 (4,974)	−1,192 (−177)	1.78/2.57	0.04/0.03	−31,165 (4,631)	−42,905 (−6,375)
**Scenario 3: Starting age = 65 years old**						
Standard treatment	24,984 (3,712)		1.86/2.51			
Vaccine + standard treatment	25,073 (3,726)	89 (13)	1.89/2.53	0.02/0.03	3,659 (544)	3,266 (485)

CNY, Chinese Yuan; QALY, quality-adjusted life year; LY, life year; HHF, hospitalization for heart failure; UEBMI, urban employee basic medical insurance.

Scenario analyses across various conditions yielded consistent results. In scenarios with the highest influenza vaccine costs or a starting age set at 65, the ICER remained below 85,698 CNY (12,734 USD) per QALY. When alternative HHF rates were considered, the influenza vaccine showed reduced costs and increased effectiveness (Table 3).

### Sensitivity analysis and scenario analysis

**Figure 2:**
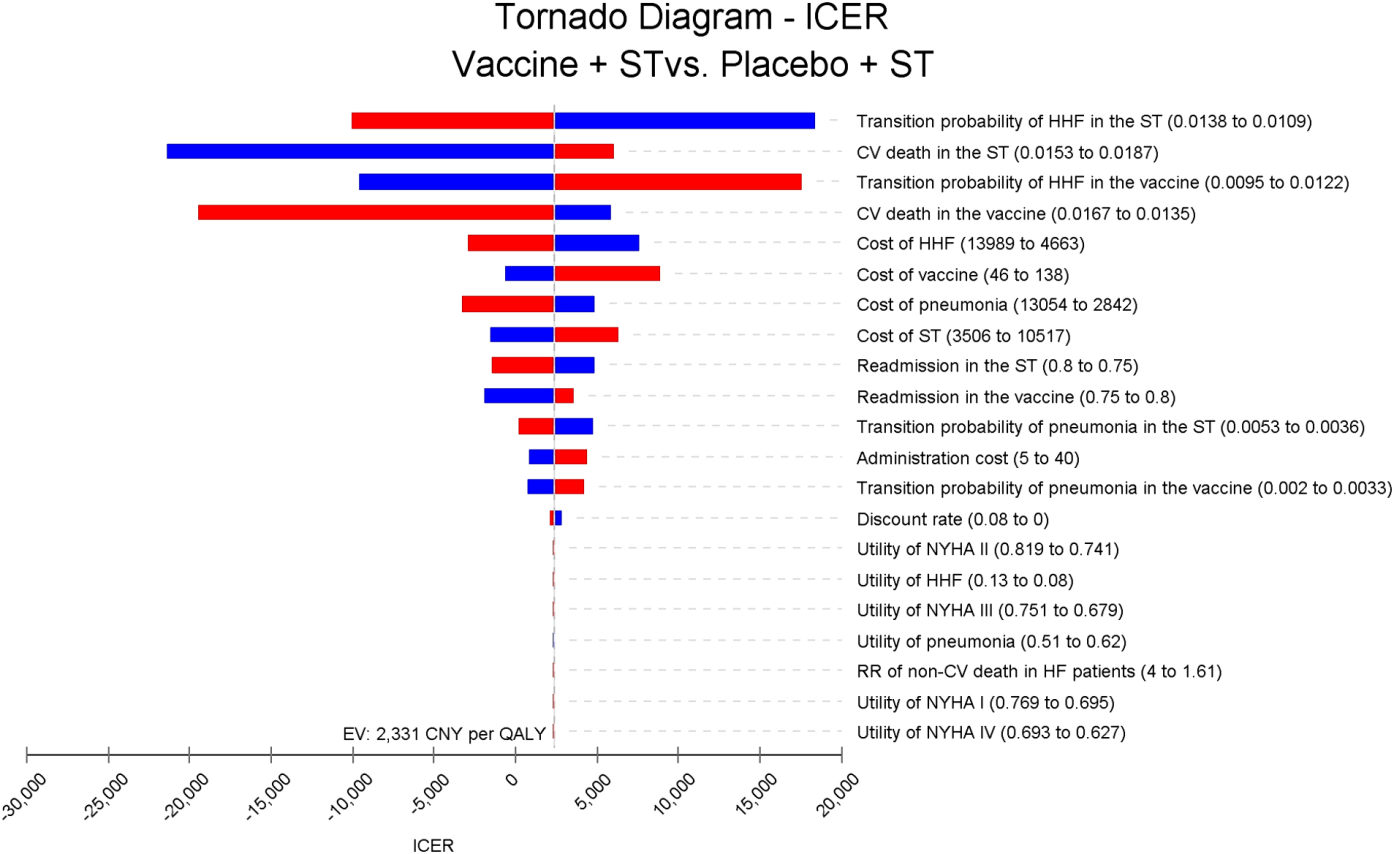
Tornado diagram for ICER of vaccine compared to placebo in Chinese HF patients. The disparities in HHF and CV death rates between groups have the most significant impact on the ICER, but do not exceed 20,000 Chinese Yuan (2,972 USD) per quality-adjusted life year. ICER, incremental cost-effectiveness ratio; ST, standard treatment; HHF, hospitalization for heart failure; CV, cardiovascular; NYHA, New York Heart Association.

One-way sensitivity analysis revealed that discrepancies in rates of HHF and CV death between groups had the most significant impact on the ICER, but none of the input parameters resulted in an ICER exceeding the WTP threshold of 85,698 CNY (12,734 USD) per QALY (Figure 2).

**Figure 3:**
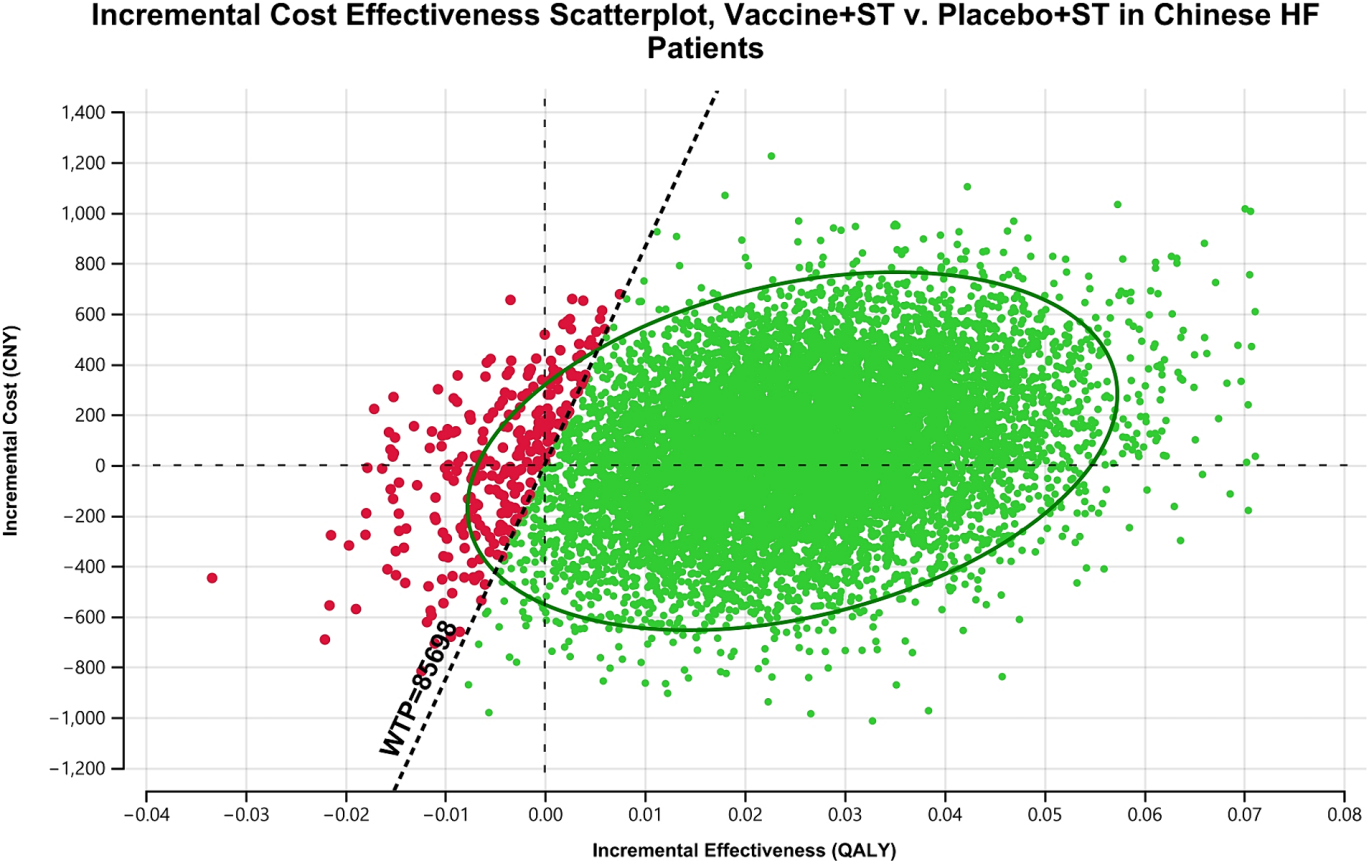
Scatterplot analysis of incremental cost effectiveness: influenza vaccine plus standard treatment vs. placebo plus standard treatment in Chinese HF patients. In most occasions, influenza vaccine is dominant or highly cost-effective compared with placebo. ST, standard treatment; HF, heart failure; CNY, Chinese Yuan; WTP, willingness-to-pay; QALY, quality-adjusted life year.

**Figure 4:**
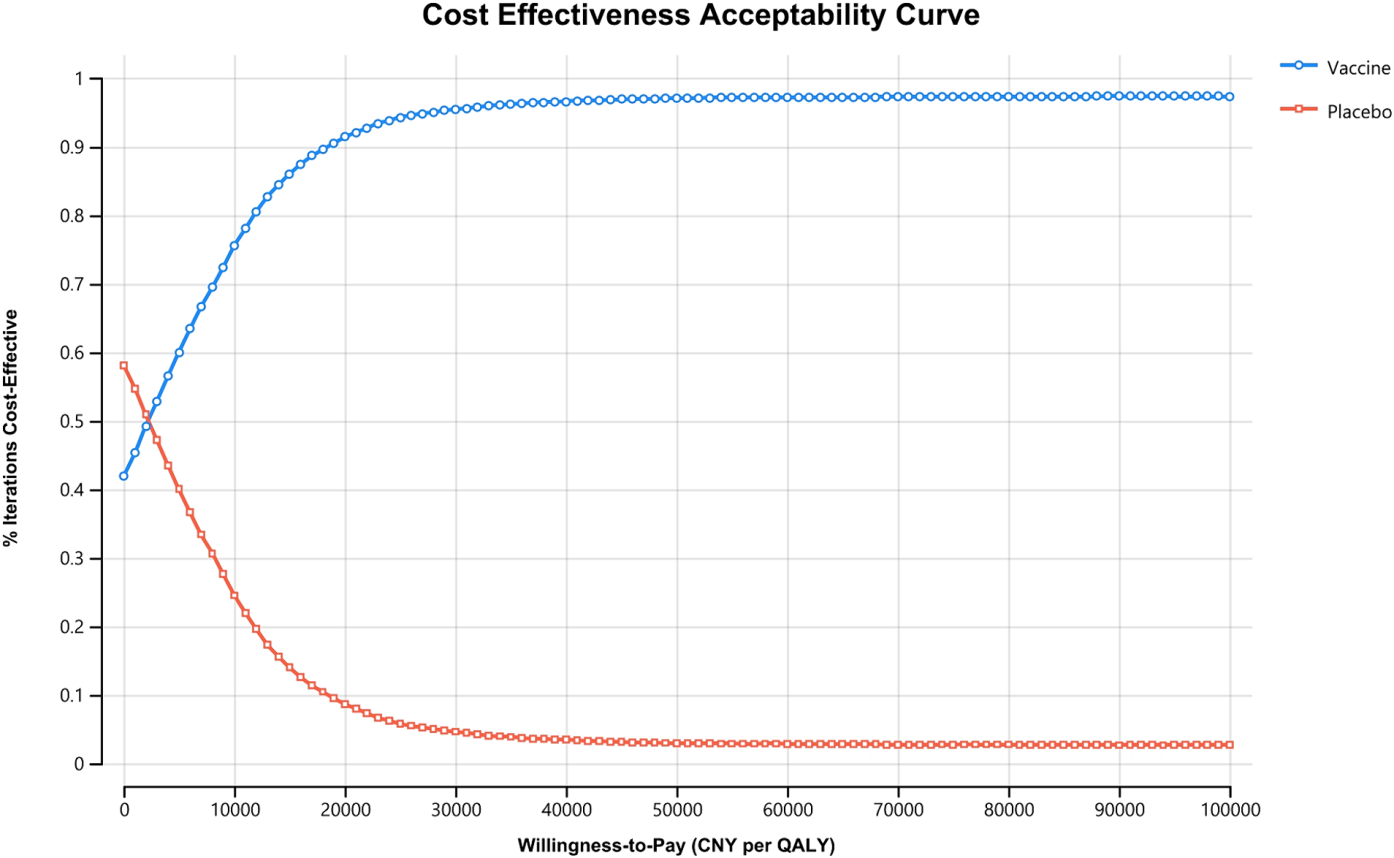
Cost effectiveness acceptability curve. The WTP threshold where vaccine is more acceptable than standard treatment in Chinese HF patients falls below 5,000 CNY (743 USD) per QALY, and vaccine is more acceptable under the current willingness-to-pay threshold of 85,698 CNY (12,734 USD) per QALY. CNY, Chinese Yuan; QALY, quality-adjusted life year.

Results from the PSA showed that administering the vaccine to Chinese HF patients alongside standard treatment was dominant in 39.9% of cases and highly cost-effective in 56.54% of scenarios. Overall, the vaccine was considered highly cost-effective in at least 96.44% of scenarios (Figure 3). The acceptability curve indicated that at the current WTP threshold of 85,698 CNY (12,734 USD) per QALY, vaccine administration had over 90% acceptability, while standard treatment had less than 10% acceptability (Figure 4).

## Discussion

In this study, the ICER of the influenza vaccine compared to a placebo for Chinese patients with HF was calculated at 2,331 CNY (346 USD) per QALY [2,080 CNY (309 USD) per life year], falling below the per capita GDP threshold of 85,698 CNY (12,734 USD) per QALY. Additionally, one-way sensitivity analysis revealed that varying parameters within specified ranges did not yield an ICER surpassing the WTP threshold. Moreover, PSA results indicated that incorporating the vaccine into standard HF treatment was highly cost-effective in 96.44% of scenarios. These consistent findings across sensitivity analyses underscore the robustness of this study and underscore the influenza vaccine’s status as a highly cost-effective intervention for Chinese HF patients.

In the IVVE trial, although the influenza vaccine did not significantly reduce adverse vascular events throughout the entire trial period, it does not negate the cost-effectiveness of vaccination in this economic evaluation. This could be attributed to several reasons: Firstly, in the original study, nearly all outcomes (including all-cause death, CV death, non-fatal myocardial infarction, non-fatal stroke, all-cause hospitalization, HHF, and pneumonia) except for non-fatal stroke were lower in the influenza vaccine group compared to the placebo group, with only all-cause hospitalization and pneumonia showing statistical significance. These observed synergistic effects may enhance vaccine effectiveness. Secondly, when considering only periods during peak influenza circulation, a significant decrease in all-cause death, CV death, and pneumonia was observed in the vaccine group. On one hand, the reduction of CV events and pneumonia decreased the related costs associated with the vaccine; on the other hand, this reduction also improved effectiveness. These factors collectively contribute to the cost-effectiveness of the influenza vaccine in Chinese HF patients.

Furthermore, it should be noted that the influenza vaccine used in the IVVE trial was a TIV, consisting of two strains of influenza A virus (H1N1 and H3N2) and one lineage of influenza B virus (Yamagata or Victoria). The production of this vaccine was undertaken by Sanofi Pasteur Biologics Co., Ltd. at a cost of 75 CNY (11 USD) per dose. Currently, QIV which included an additional strain of influenza B virus absent from TIV, have demonstrated superior protection and paralleled adverse effects globally (26, 27). Similarly, QIV is recommended by The Chinese Center for Disease Control and Prevention, and likely to be more cost-effective for the elder in China (28). And the QIV produced by Sanofi Pasteur incurs higher cost at 138 CNY (21 USD) per dose. As depicted in scenario 1, the highest price was set at 138 CNY (21 USD) per dose, which currently stands as the most expensive TIV available in China, resulting in an ICER with 8,869 CNY (1,318 USD) per QALY [7,914 CNY (1,176 USD) per life year], lower than WTP. Therefore, if using QIV under current circumstances in China, vaccination is also highly cost-effective even with higher cost.

Currently, the influenza vaccination rate in China was not optimistic. First, vaccination policies have a significant impact on vaccination rates. For instance, in the United States, universal vaccination is recommended for all individuals (29), whereas in most countries, it is only advised for those susceptible to influenza. In China, high-risk groups are defined as individuals aged 60 and above, children under 5 years old, pregnant women, and people with chronic illnesses. In fact, a Chinese national cross-sectional survey conducted in 2014–2015, encompassing a sample of 74,484 individuals aged over 40 years old, revealed that the influenza vaccination rate among individuals with chronic diseases was merely 4.0% (30). Secondly, there were significant variations in vaccination rates across different regions within China, which were caused by economic gap. For instance, the eastern region exhibited a higher vaccination rate compared to the western region (26.1% vs. 6.7%) (31, 32). In addition, as influenza vaccinations are predominantly not covered by mandatory health insurance schemes, local reimbursement ratio could also influence the vaccination rate (33). Such as Shenzhen city, the government fully covered the reimbursement for influenza vaccination in elder above 60 years old; however, in Guangzhou, a similarly developed city, reimbursement was funded through the surplus of Basic Social Medical Insurance for Urban Employees and the vaccination rate was lower. At the same time, relevant medical authorities have also implemented novel measures to bolster vaccination rates. A study has revealed that video-based education represents an effective and feasible approach for improving older individuals’ willingness and uptake of influenza vaccination (34). And interestingly, a pragmatic trial has suggested that a pay-it-forward intervention, which provides complimentary influenza vaccines along with an opportunity to contribute financially toward supporting the immunization of other individuals, could augment influenza uptake (35).

The prevalence of HF patients in China is substantial, imposing a significant burden. It is worth noting that there is a dearth of cost–benefit analyses pertaining to vaccination for HF patients in China. Meanwhile, several studies have conducted cost–benefit analyses on vaccination for older adult individuals in China (36, 37), yielding similar outcomes. Given the considerable overlap between the HF and older adult populations, it could be inferred that vaccination may be cost-effective in HF patients and exhibit enhanced efficacy, which was consistent with our findings. Overall, vaccination is a highly cost-effective intervention for HF patients, and recommended for widespread adoption among the population. Despite the gradual increase in vaccination rates in China over the past years, it remains a formidable challenge for relevant departments to augment vaccination coverage to align with that of developed nations, necessitating further concerted efforts.

This study has several limitations. Firstly, the study employed a simple Markov model based on the IVVE trial. This model considered vaccination as a static process and did not account for varying vaccination rates, timing, herd immunity effects, or adherence to vaccination schedules. Secondly, despite being a multicenter, randomized, double-blind, placebo-controlled trial, the IVVE trial recruited participants from various countries and regions, including Asia. Therefore, the participants in the IVVE trial may not fully represent Chinese patients with HF. Moreover, adverse events in the vaccination group and the control group were also not taken into account, although both groups shared comparable and extremely low incidence of adverse events. Nevertheless, we still hold the belief that this research is useful for policymakers, providing stable and conservative results.

## Conclusion

The findings of this study indicate that influenza vaccination is likely to be a highly cost-effective preventive measure for Chinese HF patients, thus warranting its widespread adoption among this patient population.

## Data availability statement

The raw data supporting the conclusions of this article will be made available by the authors, without undue reservation.

## Author contributions

MZ: Writing – review & editing, Writing – original draft. FL: Software, Methodology, Investigation, Formal analysis, Data curation, Writing – review & editing. LW: Writing – review & editing, Funding acquisition, Conceptualization, Methodology, Investigation, Formal analysis, Data curation. DC: Writing – review & editing, Supervision, Resources, Methodology, Funding acquisition, Data curation, Conceptualization.
