# Supplementary material for: Influenza vaccination for heart failure patients: a cost-effectiveness analysis from the perspective of Chinese healthcare system
Source: Front Public Health. 2024 Aug 9;12:1348207. doi: 10.3389/fpubh.2024.1348207 (PMC11341488; doi:10.3389/fpubh.2024.1348207)
Supplement: Supplementary file 1 [file Data_Sheet_1.doc]

**Supplementary materials**

**sTable 1:** **Baseline Characteristics of Heart Failure Patients in the IVVE Trial and China Hypertension Survey**

| **Variables** | **IVVE trial** | **China Hypertension Survey** |
| --- | --- | --- |
| Age | 57.2 ± 15.3 | 63.9 ± 13.2 |
| Mean heart rate, beats per min | 80.3 ± 15.0 | 77.8 ± 12.4 |
| Mean systolic blood pressure, mm Hg | 125.7 ± 23.7 | 137.2 ± 22.3 |
| Female, % | 51.4 | 45.8 |
| Left ventricular function | | |
| >50%, % | 22.6 | / |
| <50%, % | 72.8 | 69.6 |
| Unknown or missing, % | 4.6 | / |
| Hypertension, % | 64.9 | 55.3 |
| Type 2 diabetes, % | 22.6 | 18.5 |
| Atrial fibrillation, % | 10.3 | 12.1 |

**sTable 2:** Calculation methods for transition probabilities

| Parameters | Events  (E) | Total  (T) | Incidence rate  (R=E/T) | Period number  (N) | 3-month rate  (r=-ln(1–R)/N) | Transition probability  (P=1–exp(-r)) |
| --- | --- | --- | --- | --- | --- | --- |
| HHF in vaccine | 245 | 2560 | 0.095703125 | 9.2 | 0.010934519 | 0.010874954 |
| HHF in ST | 277 | 2569 | 0.107824056 | 9.2 | 0.012401296 | 0.012324716 |
| CV death in vaccine | 334 | 2560 | 0.13046875 | 9.2 | 0.015195761 | 0.015080888 |
| CV death in ST | 374 | 2569 | 0.145581938 | 9.2 | 0.017101595 | 0.016956193 |
| Pneumonia in vaccine | 61 | 2560 | 0.023828125 | 9.2 | 0.00262137 | 0.002617937 |
| Pneumonia in ST | 104 | 2569 | 0.040482678 | 9.2 | 0.004491838 | 0.004481765 |
